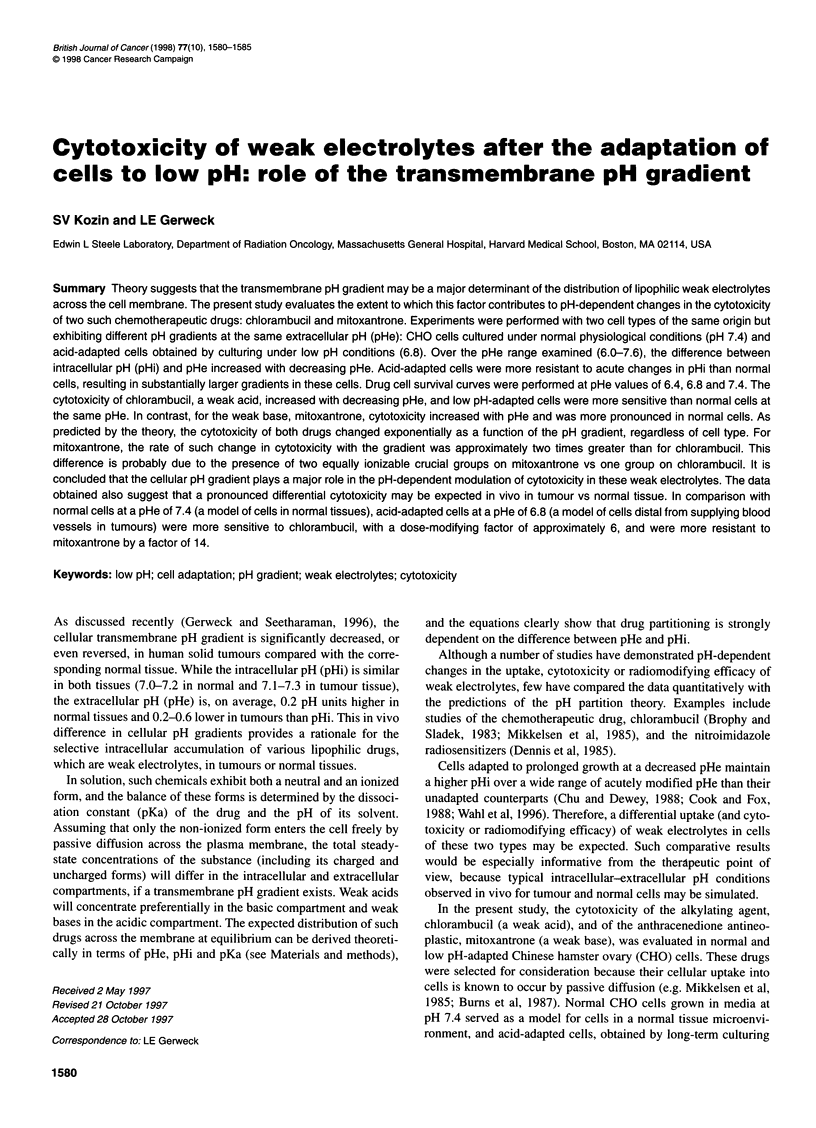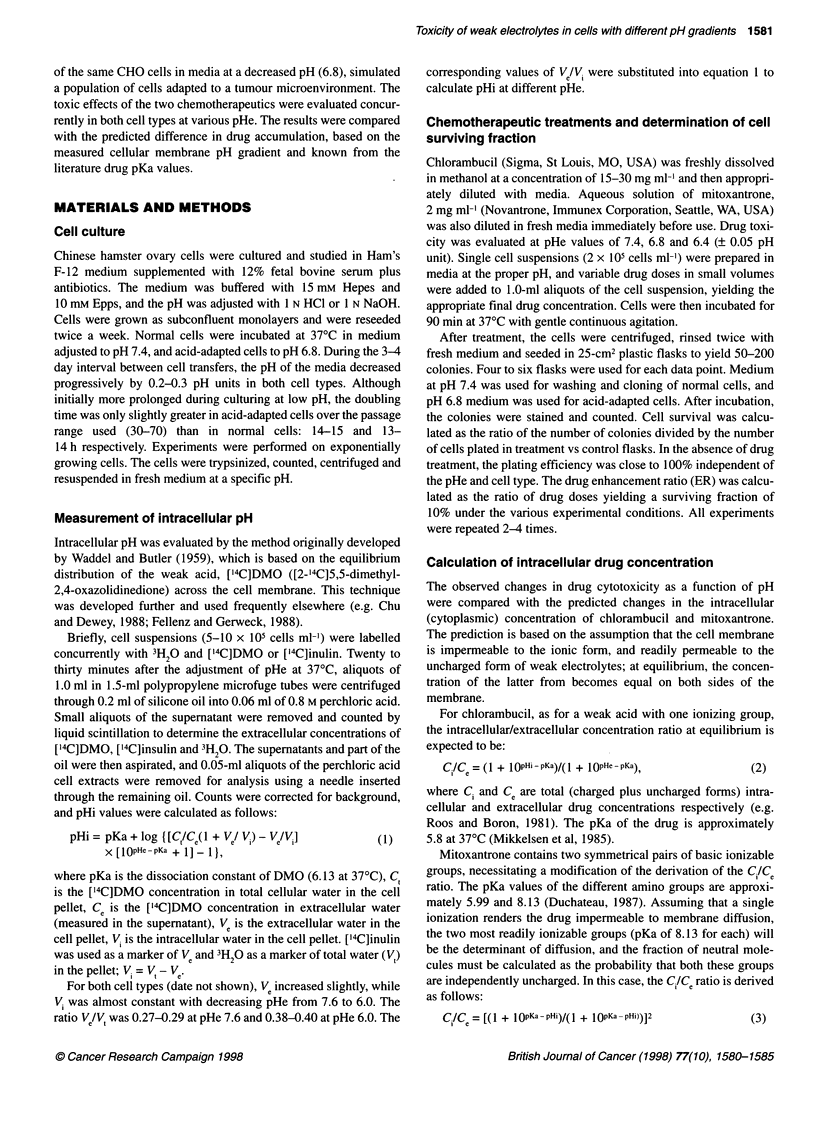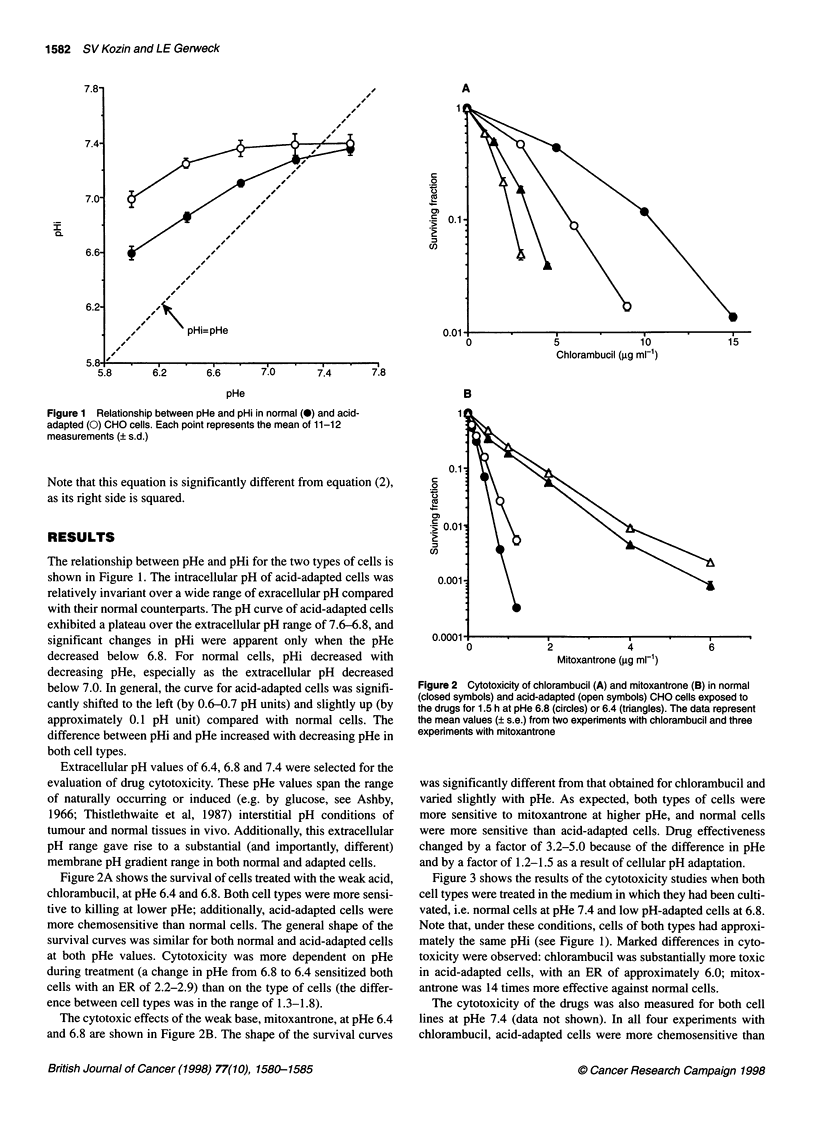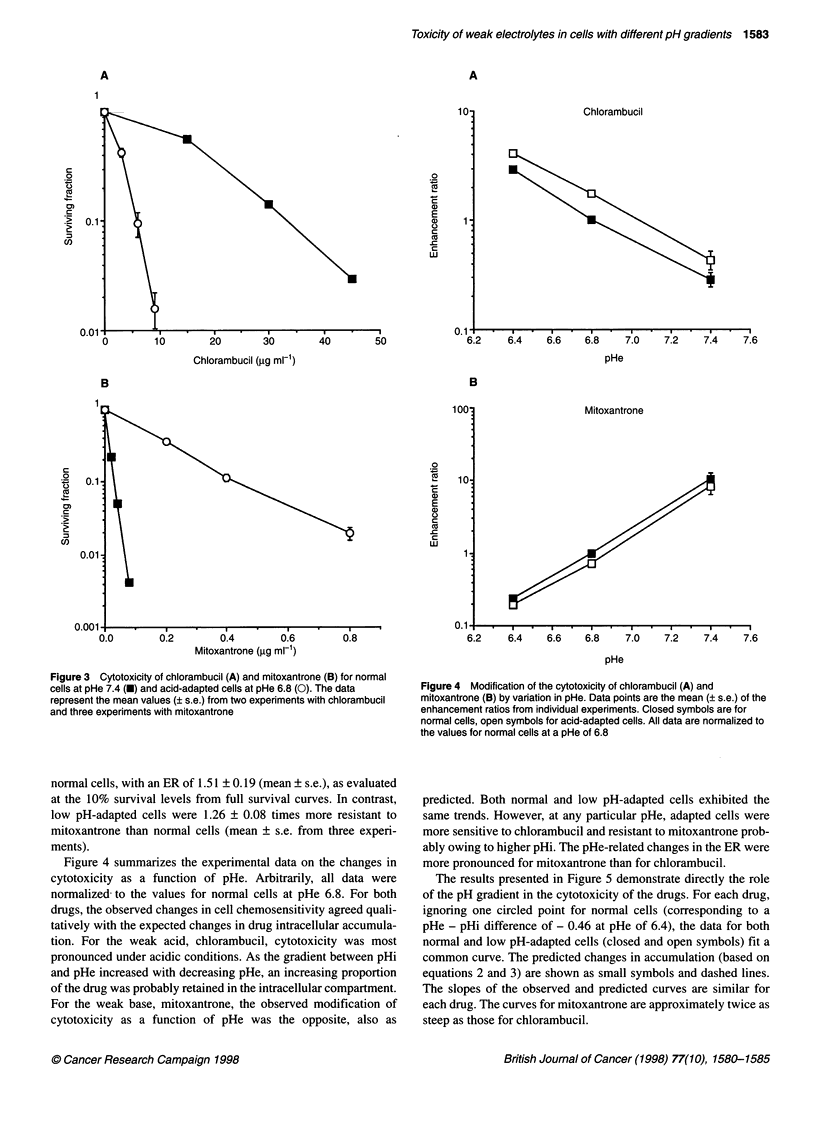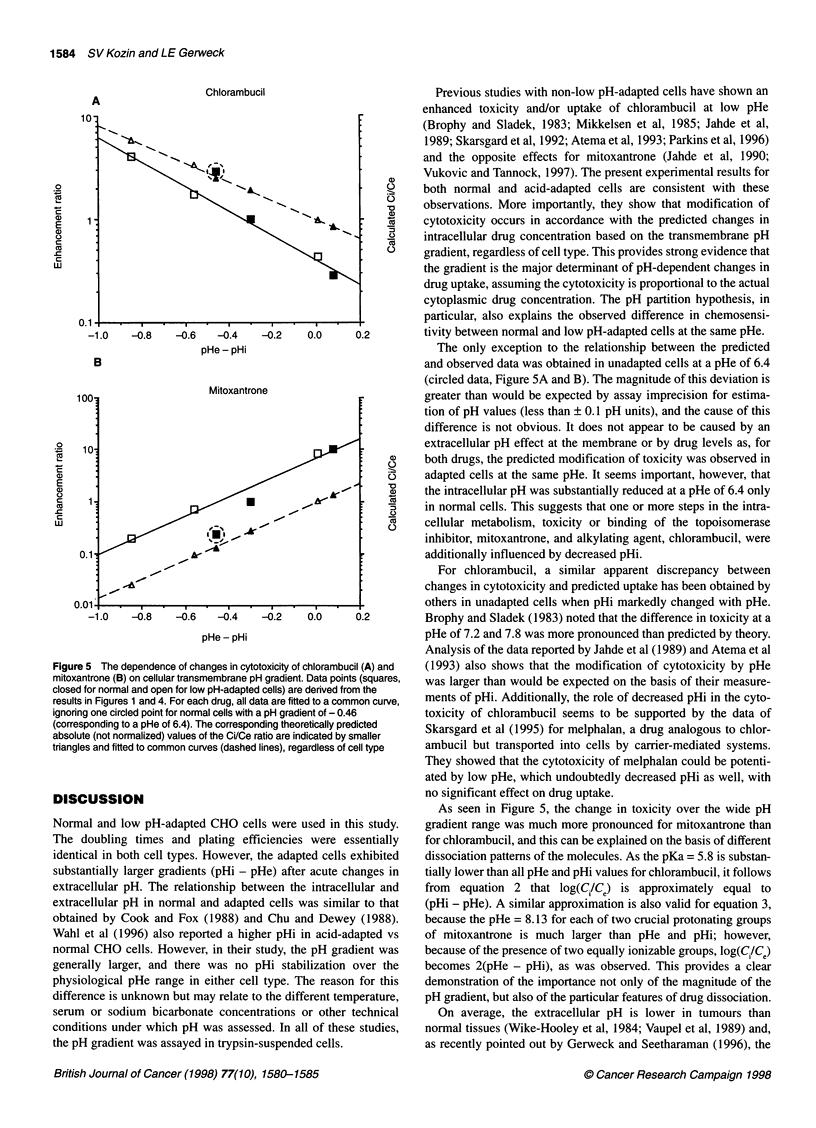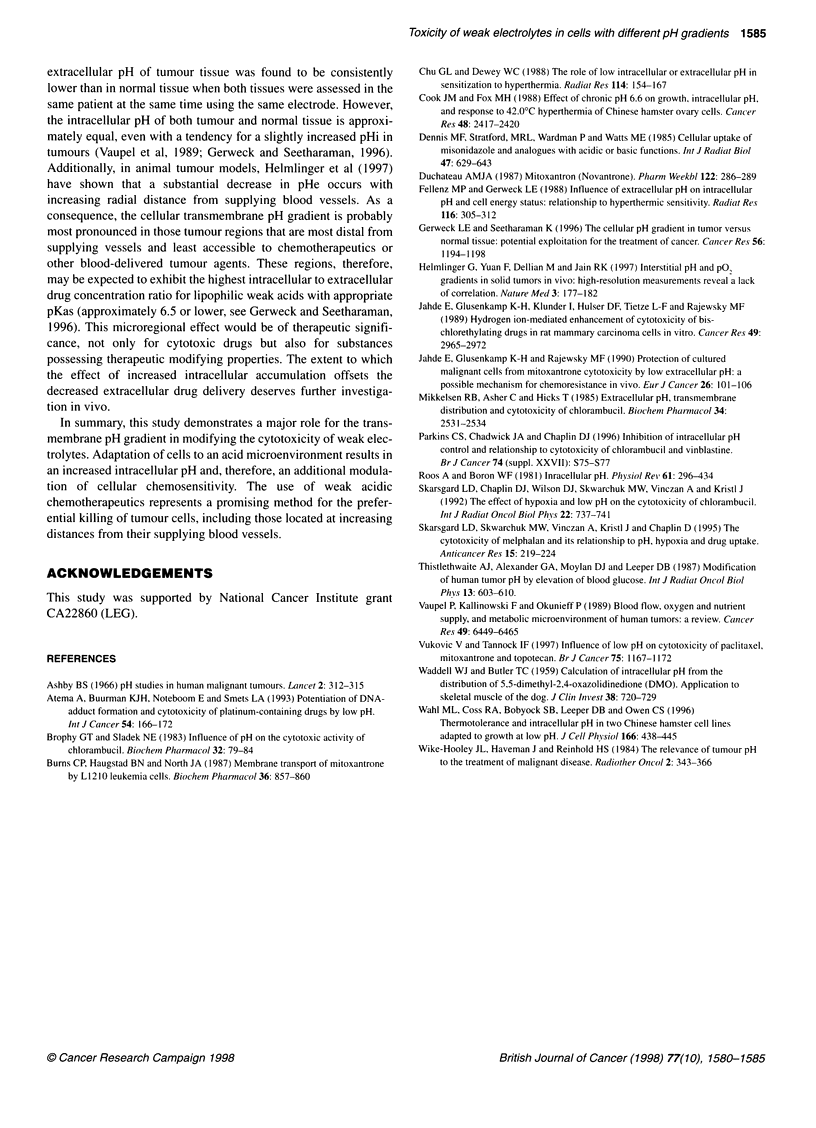# Cytotoxicity of weak electrolytes after the adaptation of cells to low pH: role of the transmembrane pH gradient.

**DOI:** 10.1038/bjc.1998.260

**Published:** 1998-05

**Authors:** S. V. Kozin, L. E. Gerweck

**Affiliations:** Edwin L Steele Laboratory, Department of Radiation Oncology, Massachusetts General Hospital, Harvard Medical School, Boston 02114, USA.

## Abstract

Theory suggests that the transmembrane pH gradient may be a major determinant of the distribution of lipophilic weak electrolytes across the cell membrane. The present study evaluates the extent to which this factor contributes to pH-dependent changes in the cytotoxicity of two such chemotherapeutic drugs: chlorambucil and mitoxantrone. Experiments were performed with two cell types of the same origin but exhibiting different pH gradients at the same extracellular pH (pHe): CHO cells cultured under normal physiological conditions (pH 7.4) and acid-adapted cells obtained by culturing under low pH conditions (6.8). Over the pHe range examined (6.0-7.6), the difference between intracellular pH (pHi) and pHe increased with decreasing pHe. Acid-adapted cells were more resistant to acute changes in pHi than normal cells, resulting in substantially larger gradients in these cells. Drug cell survival curves were performed at pHe values of 6.4, 6.8 and 7.4. The cytotoxicity of chlorambucil, a weak acid, increased with decreasing pHe, and low pH-adapted cells were more sensitive than normal cells at the same pHe. In contrast, for the weak base, mitoxantrone, cytotoxicity increased with pHe and was more pronounced in normal cells. As predicted by the theory, the cytotoxicity of both drugs changed exponentially as a function of the pH gradient, regardless of cell type. For mitoxantrone, the rate of such change in cytotoxicity with the gradient was approximately two times greater than for chlorambucil. This difference is probably due to the presence of two equally ionizable crucial groups on mitoxantrone vs one group on chlorambucil. It is concluded that the cellular pH gradient plays a major role in the pH-dependent modulation of cytotoxicity in these weak electrolytes. The data obtained also suggest that a pronounced differential cytotoxicity may be expected in vivo in tumour vs normal tissue. In comparison with normal cells at a pHe of 7.4 (a model of cells in normal tissues), acid-adapted cells at a pHe of 6.8 (a model of cells distal from supplying blood vessels in tumours) were more sensitive to chlorambucil, with a dose-modifying factor of approximately 6, and were more resistant to mitoxantrone by a factor of 14.


British Journal of Cancer (1998) 77(10), 1580–1585
© 1998 Cancer Research Campaign

### Cytotoxicity of weak electrolytes after the adaptation of cells to low pH: role of the transmembrane pH gradient

**SV Kozin and LE Gerweck**

Edwin L Steele Laboratory, Department of Radiation Oncology, Massachusetts General Hospital, Harvard Medical School, Boston, MA 02114, USA

**Summary** Theory suggests that the transmembrane pH gradient may be a major determinant of the distribution of lipophilic weak electrolytes across the cell membrane. The present study evaluates the extent to which this factor contributes to pH-dependent changes in the cytotoxicity of two such chemotherapeutic drugs: chlorambucil and mitoxantrone. Experiments were performed with two cell types of the same origin but exhibiting different pH gradients at the same extracellular pH (pHe): CHO cells cultured under normal physiological conditions (pH 7.4) and acid-adapted cells obtained by culturing under low pH conditions (6.8). Over the pHe range examined (6.0–7.6), the difference between intracellular pH (pHi) and pHe increased with decreasing pHe. Acid-adapted cells were more resistant to acute changes in pHi than normal cells, resulting in substantially larger gradients in these cells. Drug cell survival curves were performed at pHe values of 6.4, 6.8 and 7.4. The cytotoxicity of chlorambucil, a weak acid, increased with decreasing pHe, and low pH-adapted cells were more sensitive than normal cells at the same pHe. In contrast, for the weak base, mitoxantrone, cytotoxicity increased with pHe and was more pronounced in normal cells. As predicted by the theory, the cytotoxicity of both drugs changed exponentially as a function of the pH gradient, regardless of cell type. For mitoxantrone, the rate of such change in cytotoxicity with the gradient was approximately two times greater than for chlorambucil. This difference is probably due to the presence of two equally ionizable crucial groups on mitoxantrone vs one group on chlorambucil. It is concluded that the cellular pH gradient plays a major role in the pH-dependent modulation of cytotoxicity in these weak electrolytes. The data obtained also suggest that a pronounced differential cytotoxicity may be expected in vivo in tumour vs normal tissue. In comparison with normal cells at a pHe of 7.4 (a model of cells in normal tissues), acid-adapted cells at a pHe of 6.8 (a model of cells distal from supplying blood vessels in tumours) were more sensitive to chlorambucil, with a dose-modifying factor of approximately 6, and were more resistant to mitoxantrone by a factor of 14.

**Keywords:** low pH; cell adaptation; pH gradient; weak electrolytes; cytotoxicity

As discussed recently (Gerweck and Seetharaman, 1996), the cellular transmembrane pH gradient is significantly decreased, or even reversed, in human solid tumours compared with the corresponding normal tissue. While the intracellular pH (pHi) is similar in both tissues (7.0–7.2 in normal and 7.1–7.3 in tumour tissue), the extracellular pH (pHe) is, on average, 0.2 pH units higher in normal tissues and 0.2–0.6 lower in tumours than pHi. This in vivo difference in cellular pH gradients provides a rationale for the selective intracellular accumulation of various lipophilic drugs, which are weak electrolytes, in tumours or normal tissues.

In solution, such chemicals exhibit both a neutral and an ionized form, and the balance of these forms is determined by the dissociation constant (pKa) of the drug and the pH of its solvent. Assuming that only the non-ionized form enters the cell freely by passive diffusion across the plasma membrane, the total steady-state concentrations of the substance (including its charged and uncharged forms) will differ in the intracellular and extracellular compartments, if a transmembrane pH gradient exists. Weak acids will concentrate preferentially in the basic compartment and weak bases in the acidic compartment. The expected distribution of such drugs across the membrane at equilibrium can be derived theoretically in terms of pHe, pHi and pKa (see Materials and methods), and the equations clearly show that drug partitioning is strongly dependent on the difference between pHe and pHi.

Although a number of studies have demonstrated pH-dependent changes in the uptake, cytotoxicity or radiomodifying efficacy of weak electrolytes, few have compared the data quantitatively with the predictions of the pH partition theory. Examples include studies of the chemotherapeutic drug, chlorambucil (Brophy and Sladek, 1983; Mikkelsen et al, 1985), and the nitroimidazole radiosensitizers (Dennis et al, 1985).

Cells adapted to prolonged growth at a decreased pHe maintain a higher pHi over a wide range of acutely modified pHe than their unadapted counterparts (Chu and Dewey, 1988; Cook and Fox, 1988; Wahl et al, 1996). Therefore, a differential uptake (and cytotoxicity or radiomodifying efficacy) of weak electrolytes in cells of these two types may be expected. Such comparative results would be especially informative from the therapeutic point of view, because typical intracellular–extracellular pH conditions observed in vivo for tumour and normal cells may be simulated.

In the present study, the cytotoxicity of the alkylating agent, chlorambucil (a weak acid), and of the anthracenedione antineoplastic, mitoxantrone (a weak base), was evaluated in normal and low pH-adapted Chinese hamster ovary (CHO) cells. These drugs were selected for consideration because their cellular uptake into cells is known to occur by passive diffusion (e.g. Mikkelsen et al, 1985; Burns et al, 1987). Normal CHO cells grown in media at pH 7.4 served as a model for cells in a normal tissue microenvironment, and acid-adapted cells, obtained by long-term culturing

*Received 2 May 1997*
*Revised 21 October 1997*
*Accepted 28 October 1997*

*Correspondence to:* LE Gerweck of the same CHO cells in media at a decreased pH (6.8), simulated a population of cells adapted to a tumour microenvironment. The toxic effects of the two chemotherapeutics were evaluated concurrently in both cell types at various pHe. The results were compared with the predicted difference in drug accumulation, based on the measured cellular membrane pH gradient and known from the literature drug pKa values.

### MATERIALS AND METHODS

### Cell culture

Chinese hamster ovary cells were cultured and studied in Ham's F-12 medium supplemented with 12% fetal bovine serum plus antibiotics. The medium was buffered with 15 mM Hepes and 10 mM Epps, and the pH was adjusted with 1 N HCl or 1 N NaOH. Cells were grown as subconfluent monolayers and were reseeded twice a week. Normal cells were incubated at 37°C in medium adjusted to pH 7.4, and acid-adapted cells to pH 6.8. During the 3–4 day interval between cell transfers, the pH of the media decreased progressively by 0.2–0.3 pH units in both cell types. Although initially more prolonged during culturing at low pH, the doubling time was only slightly greater in acid-adapted cells over the passage range used (30–70) than in normal cells: 14–15 and 13–14 h respectively. Experiments were performed on exponentially growing cells. The cells were trypsinized, counted, centrifuged and resuspended in fresh medium at a specific pH.

### Measurement of intracellular pH

Intracellular pH was evaluated by the method originally developed by Waddel and Butler (1959), which is based on the equilibrium distribution of the weak acid, [$^{14}$C]DMO ([2-$^{14}$C]5,5-dimethyl-2,4-oxazolidinedione) across the cell membrane. This technique was developed further and used frequently elsewhere (e.g. Chu and Dewey, 1988; Fellenz and Gerweck, 1988).

Briefly, cell suspensions (5–10 × $10^5$ cells ml$^{-1}$) were labelled concurrently with $^3H_2O$ and [$^{14}$C]DMO or [$^{14}$C]inulin. Twenty to thirty minutes after the adjustment of pHe at 37°C, aliquots of 1.0 ml in 1.5-ml polypropylene microfuge tubes were centrifuged through 0.2 ml of silicone oil into 0.06 ml of 0.8 M perchloric acid. Small aliquots of the supernatant were removed and counted by liquid scintillation to determine the extracellular concentrations of [$^{14}$C]DMO, [$^{14}$C]insulin and $^3H_2O$. The supernatants and part of the oil were then aspirated, and 0.05-ml aliquots of the perchloric acid cell extracts were removed for analysis using a needle inserted through the remaining oil. Counts were corrected for background, and pHi values were calculated as follows:

$$\mathrm{pHi} = \mathrm{pKa} + \log\{[C_t/C_e(1 + V_e/V_i) - V_e/V_i] \times [10^{\mathrm{pHe}-\mathrm{pKa}} + 1] - 1\}, \quad (1)$$

where pKa is the dissociation constant of DMO (6.13 at 37°C), $C_t$ is the [$^{14}$C]DMO concentration in total cellular water in the cell pellet, $C_e$ is the [$^{14}$C]DMO concentration in extracellular water (measured in the supernatant), $V_e$ is the extracellular water in the cell pellet, $V_i$ is the intracellular water in the cell pellet. [$^{14}$C]inulin was used as a marker of $V_e$ and $^3H_2O$ as a marker of total water ($V_t$) in the pellet; $V_i = V_t - V_e$.

For both cell types (date not shown), $V_e$ increased slightly, while $V_i$ was almost constant with decreasing pHe from 7.6 to 6.0. The ratio $V_e/V_t$ was 0.27–0.29 at pHe 7.6 and 0.38–0.40 at pHe 6.0. The corresponding values of $V_e/V_i$ were substituted into equation 1 to calculate pHi at different pHe.

### Chemotherapeutic treatments and determination of cell surviving fraction

Chlorambucil (Sigma, St Louis, MO, USA) was freshly dissolved in methanol at a concentration of 15–30 mg ml$^{-1}$ and then appropriately diluted with media. Aqueous solution of mitoxantrone, 2 mg ml$^{-1}$ (Novantrone, Immunex Corporation, Seattle, WA, USA) was also diluted in fresh media immediately before use. Drug toxicity was evaluated at pHe values of 7.4, 6.8 and 6.4 (± 0.05 pH unit). Single cell suspensions (2 × $10^5$ cells ml$^{-1}$) were prepared in media at the proper pH, and variable drug doses in small volumes were added to 1.0-ml aliquots of the cell suspension, yielding the appropriate final drug concentration. Cells were then incubated for 90 min at 37°C with gentle continuous agitation.

After treatment, the cells were centrifuged, rinsed twice with fresh medium and seeded in 25-cm$^2$ plastic flasks to yield 50–200 colonies. Four to six flasks were used for each data point. Medium at pH 7.4 was used for washing and cloning of normal cells, and pH 6.8 medium was used for acid-adapted cells. After incubation, the colonies were stained and counted. Cell survival was calculated as the ratio of the number of colonies divided by the number of cells plated in treatment vs control flasks. In the absence of drug treatment, the plating efficiency was close to 100% independent of the pHe and cell type. The drug enhancement ratio (ER) was calculated as the ratio of drug doses yielding a surviving fraction of 10% under the various experimental conditions. All experiments were repeated 2–4 times.

### Calculation of intracellular drug concentration

The observed changes in drug cytotoxicity as a function of pH were compared with the predicted changes in the intracellular (cytoplasmic) concentration of chlorambucil and mitoxantrone. The prediction is based on the assumption that the cell membrane is impermeable to the ionic form, and readily permeable to the uncharged form of weak electrolytes; at equilibrium, the concentration of the latter from becomes equal on both sides of the membrane.

For chlorambucil, as for a weak acid with one ionizing group, the intracellular/extracellular concentration ratio at equilibrium is expected to be:

$$C_i/C_e = (1 + 10^{\mathrm{pHi}-\mathrm{pKa}})/(1 + 10^{\mathrm{pHe}-\mathrm{pKa}}), \quad (2)$$

where $C_i$ and $C_e$ are total (charged plus uncharged forms) intracellular and extracellular drug concentrations respectively (e.g. Roos and Boron, 1981). The pKa of the drug is approximately 5.8 at 37°C (Mikkelsen et al, 1985).

Mitoxantrone contains two symmetrical pairs of basic ionizable groups, necessitating a modification of the derivation of the $C_i/C_e$ ratio. The pKa values of the different amino groups are approximately 5.99 and 8.13 (Duchateau, 1987). Assuming that a single ionization renders the drug impermeable to membrane diffusion, the two most readily ionizable groups (pKa of 8.13 for each) will be the determinant of diffusion, and the fraction of neutral molecules must be calculated as the probability that both these groups are independently uncharged. In this case, the $C_i/C_e$ ratio is derived as follows:

$$C_i/C_e = [(1 + 10^{\mathrm{pKa}-\mathrm{pHi}})/(1 + 10^{\mathrm{pKa}-\mathrm{pHi}})]^2 \quad (3)$$

**Figure 1** Relationship between pHe and pHi in normal (●) and acid-adapted (○) CHO cells. Each point represents the mean of 11–12 measurements (± s.d.)

Note that this equation is significantly different from equation (2), as its right side is squared.

### RESULTS

The relationship between pHe and pHi for the two types of cells is shown in Figure 1. The intracellular pH of acid-adapted cells was relatively invariant over a wide range of exracellular pH compared with their normal counterparts. The pH curve of acid-adapted cells exhibited a plateau over the extracellular pH range of 7.6–6.8, and significant changes in pHi were apparent only when the pHe decreased below 6.8. For normal cells, pHi decreased with decreasing pHe, especially as the extracellular pH decreased below 7.0. In general, the curve for acid-adapted cells was significantly shifted to the left (by 0.6–0.7 pH units) and slightly up (by approximately 0.1 pH unit) compared with normal cells. The difference between pHi and pHe increased with decreasing pHe in both cell types.

Extracellular pH values of 6.4, 6.8 and 7.4 were selected for the evaluation of drug cytotoxicity. These pHe values span the range of naturally occurring or induced (e.g. by glucose, see Ashby, 1966; Thistlethwaite et al, 1987) interstitial pH conditions of tumour and normal tissues in vivo. Additionally, this extracellular pH range gave rise to a substantial (and importantly, different) membrane pH gradient range in both normal and adapted cells.

Figure 2A shows the survival of cells treated with the weak acid, chlorambucil, at pHe 6.4 and 6.8. Both cell types were more sensitive to killing at lower pHe; additionally, acid-adapted cells were more chemosensitive than normal cells. The general shape of the survival curves was similar for both normal and acid-adapted cells at both pHe values. Cytotoxicity was more dependent on pHe during treatment (a change in pHe from 6.8 to 6.4 sensitized both cells with an ER of 2.2–2.9) than on the type of cells (the difference between cell types was in the range of 1.3–1.8).

The cytotoxic effects of the weak base, mitoxantrone, at pHe 6.4 and 6.8 are shown in Figure 2B. The shape of the survival curves

**Figure 2** Cytotoxicity of chlorambucil (**A**) and mitoxantrone (**B**) in normal (closed symbols) and acid-adapted (open symbols) CHO cells exposed to the drugs for 1.5 h at pHe 6.8 (circles) or 6.4 (triangles). The data represent the mean values (± s.e.) from two experiments with chlorambucil and three experiments with mitoxantrone was significantly different from that obtained for chlorambucil and varied slightly with pHe. As expected, both types of cells were more sensitive to mitoxantrone at higher pHe, and normal cells were more sensitive than acid-adapted cells. Drug effectiveness changed by a factor of 3.2–5.0 because of the difference in pHe and by a factor of 1.2–1.5 as a result of cellular pH adaptation.

Figure 3 shows the results of the cytotoxicity studies when both cell types were treated in the medium in which they had been cultivated, i.e. normal cells at pHe 7.4 and low pH-adapted cells at 6.8. Note that, under these conditions, cells of both types had approximately the same pHi (see Figure 1). Marked differences in cytotoxicity were observed: chlorambucil was substantially more toxic in acid-adapted cells, with an ER of approximately 6.0; mitoxantrone was 14 times more effective against normal cells.

The cytotoxicity of the drugs was also measured for both cell lines at pHe 7.4 (data not shown). In all four experiments with chlorambucil, acid-adapted cells were more chemosensitive than

© Cancer Research Campaign 1998

**Figure 3** Cytotoxicity of chlorambucil (**A**) and mitoxantrone (**B**) for normal cells at pHe 7.4 (■) and acid-adapted cells at pHe 6.8 (○). The data represent the mean values (± s.e.) from two experiments with chlorambucil and three experiments with mitoxantrone

**Figure 4** Modification of the cytotoxicity of chlorambucil (**A**) and mitoxantrone (**B**) by variation in pHe. Data points are the mean (± s.e.) of the enhancement ratios from individual experiments. Closed symbols are for normal cells, open symbols for acid-adapted cells. All data are normalized to the values for normal cells at a pHe of 6.8

normal cells, with an ER of 1.51 ± 0.19 (mean ± s.e.), as evaluated at the 10% survival levels from full survival curves. In contrast, low pH-adapted cells were 1.26 ± 0.08 times more resistant to mitoxantrone than normal cells (mean ± s.e. from three experiments).

Figure 4 summarizes the experimental data on the changes in cytotoxicity as a function of pHe. Arbitrarily, all data were normalized to the values for normal cells at pHe 6.8. For both drugs, the observed changes in cell chemosensitivity agreed qualitatively with the expected changes in drug intracellular accumulation. For the weak acid, chlorambucil, cytotoxicity was most pronounced under acidic conditions. As the gradient between pHi and pHe increased with decreasing pHe, an increasing proportion of the drug was probably retained in the intracellular compartment. For the weak base, mitoxantrone, the observed modification of cytotoxicity as a function of pHe was the opposite, also as predicted. Both normal and low pH-adapted cells exhibited the same trends. However, at any particular pHe, adapted cells were more sensitive to chlorambucil and resistant to mitoxantrone probably owing to higher pHi. The pHe-related changes in the ER were more pronounced for mitoxantrone than for chlorambucil.

The results presented in Figure 5 demonstrate directly the role of the pH gradient in the cytotoxicity of the drugs. For each drug, ignoring one circled point for normal cells (corresponding to a pHe – pHi difference of – 0.46 at pHe of 6.4), the data for both normal and low pH-adapted cells (closed and open symbols) fit a common curve. The predicted changes in accumulation (based on equations 2 and 3) are shown as small symbols and dashed lines. The slopes of the observed and predicted curves are similar for each drug. The curves for mitoxantrone are approximately twice as steep as those for chlorambucil.

© Cancer Research Campaign 1998

**Figure 5** The dependence of changes in cytotoxicity of chlorambucil (**A**) and mitoxantrone (**B**) on cellular transmembrane pH gradient. Data points (squares, closed for normal and open for low pH-adapted cells) are derived from the results in Figures 1 and 4. For each drug, all data are fitted to a common curve, ignoring one circled point for normal cells with a pH gradient of – 0.46 (corresponding to a pHe of 6.4). The corresponding theoretically predicted absolute (not normalized) values of the Ci/Ce ratio are indicated by smaller triangles and fitted to common curves (dashed lines), regardless of cell type

### DISCUSSION

Normal and low pH-adapted CHO cells were used in this study. The doubling times and plating efficiencies were essentially identical in both cell types. However, the adapted cells exhibited substantially larger gradients (pHi – pHe) after acute changes in extracellular pH. The relationship between the intracellular and extracellular pH in normal and adapted cells was similar to that obtained by Cook and Fox (1988) and Chu and Dewey (1988). Wahl et al (1996) also reported a higher pHi in acid-adapted vs normal CHO cells. However, in their study, the pH gradient was generally larger, and there was no pHi stabilization over the physiological pHe range in either cell type. The reason for this difference is unknown but may relate to the different temperature, serum or sodium bicarbonate concentrations or other technical conditions under which pH was assessed. In all of these studies, the pH gradient was assayed in trypsin-suspended cells.

Previous studies with non-low pH-adapted cells have shown an enhanced toxicity and/or uptake of chlorambucil at low pHe (Brophy and Sladek, 1983; Mikkelsen et al, 1985; Jahde et al, 1989; Skarsgard et al, 1992; Atema et al, 1993; Parkins et al, 1996) and the opposite effects for mitoxantrone (Jahde et al, 1990; Vukovic and Tannock, 1997). The present experimental results for both normal and acid-adapted cells are consistent with these observations. More importantly, they show that modification of cytotoxicity occurs in accordance with the predicted changes in intracellular drug concentration based on the transmembrane pH gradient, regardless of cell type. This provides strong evidence that the gradient is the major determinant of pH-dependent changes in drug uptake, assuming the cytotoxicity is proportional to the actual cytoplasmic drug concentration. The pH partition hypothesis, in particular, also explains the observed difference in chemosensitivity between normal and low pH-adapted cells at the same pHe.

The only exception to the relationship between the predicted and observed data was obtained in unadapted cells at a pHe of 6.4 (circled data, Figure 5A and B). The magnitude of this deviation is greater than would be expected by assay imprecision for estimation of pH values (less than ± 0.1 pH units), and the cause of this difference is not obvious. It does not appear to be caused by an extracellular pH effect at the membrane or by drug levels as, for both drugs, the predicted modification of toxicity was observed in adapted cells at the same pHe. It seems important, however, that the intracellular pH was substantially reduced at a pHe of 6.4 only in normal cells. This suggests that one or more steps in the intracellular metabolism, toxicity or binding of the topoisomerase inhibitor, mitoxantrone, and alkylating agent, chlorambucil, were additionally influenced by decreased pHi.

For chlorambucil, a similar apparent discrepancy between changes in cytotoxicity and predicted uptake has been obtained by others in unadapted cells when pHi markedly changed with pHe. Brophy and Sladek (1983) noted that the difference in toxicity at a pHe of 7.2 and 7.8 was more pronounced than predicted by theory. Analysis of the data reported by Jahde et al (1989) and Atema et al (1993) also shows that the modification of cytotoxicity by pHe was larger than would be expected on the basis of their measurements of pHi. Additionally, the role of decreased pHi in the cytotoxicity of chlorambucil seems to be supported by the data of Skarsgard et al (1995) for melphalan, a drug analogous to chlorambucil but transported into cells by carrier-mediated systems. They showed that the cytotoxicity of melphalan could be potentiated by low pHe, which undoubtedly decreased pHi as well, with no significant effect on drug uptake.

As seen in Figure 5, the change in toxicity over the wide pH gradient range was much more pronounced for mitoxantrone than for chlorambucil, and this can be explained on the basis of different dissociation patterns of the molecules. As the pKa = 5.8 is substantially lower than all pHe and pHi values for chlorambucil, it follows from equation 2 that $\log(C_i/C_e)$ is approximately equal to (pHi – pHe). A similar approximation is also valid for equation 3, because the pHe = 8.13 for each of two crucial protonating groups of mitoxantrone is much larger than pHe and pHi; however, because of the presence of two equally ionizable groups, $\log(C_i/C_e)$ becomes 2(pHe – pHi), as was observed. This provides a clear demonstration of the importance not only of the magnitude of the pH gradient, but also of the particular features of drug dissociation.

On average, the extracellular pH is lower in tumours than normal tissues (Wike-Hooley et al, 1984; Vaupel et al, 1989) and, as recently pointed out by Gerweck and Seetharaman (1996), the

 © Cancer Research Campaign 1998

extracellular pH of tumour tissue was found to be consistently lower than in normal tissue when both tissues were assessed in the same patient at the same time using the same electrode. However, the intracellular pH of both tumour and normal tissue is approximately equal, even with a tendency for a slightly increased pHi in tumours (Vaupel et al, 1989; Gerweck and Seetharaman, 1996). Additionally, in animal tumour models, Helmlinger et al (1997) have shown that a substantial decrease in pHe occurs with increasing radial distance from supplying blood vessels. As a consequence, the cellular transmembrane pH gradient is probably most pronounced in those tumour regions that are most distal from supplying vessels and least accessible to chemotherapeutics or other blood-delivered tumour agents. These regions, therefore, may be expected to exhibit the highest intracellular to extracellular drug concentration ratio for lipophilic weak acids with appropriate pKas (approximately 6.5 or lower, see Gerweck and Seetharaman, 1996). This microregional effect would be of therapeutic significance, not only for cytotoxic drugs but also for substances possessing therapeutic modifying properties. The extent to which the effect of increased intracellular accumulation offsets the decreased extracellular drug delivery deserves further investigation in vivo.

In summary, this study demonstrates a major role for the transmembrane pH gradient in modifying the cytotoxicity of weak electrolytes. Adaptation of cells to an acid microenvironment results in an increased intracellular pH and, therefore, an additional modulation of cellular chemosensitivity. The use of weak acidic chemotherapeutics represents a promising method for the preferential killing of tumour cells, including those located at increasing distances from their supplying blood vessels.

### ACKNOWLEDGEMENTS

This study was supported by National Cancer Institute grant CA22860 (LEG).

### REFERENCES

Ashby BS (1966) pH studies in human malignant tumours. *Lancet* **2**: 312–315

Atema A, Buurman KJH, Noteboom E and Smets LA (1993) Potentiation of DNA-adduct formation and cytotoxicity of platinum-containing drugs by low pH. *Int J Cancer* **54**: 166–172

Brophy GT and Sladek NE (1983) Influence of pH on the cytotoxic activity of chlorambucil. *Biochem Pharmacol* **32**: 79–84

Burns CP, Haugstad BN and North JA (1987) Membrane transport of mitoxantrone by L1210 leukemia cells. *Biochem Pharmacol* **36**: 857–860

Chu GL and Dewey WC (1988) The role of low intracellular or extracellular pH in sensitization to hyperthermia. *Radiat Res* **114**: 154–167

Cook JM and Fox MH (1988) Effect of chronic pH 6.6 on growth, intracellular pH, and response to 42.0°C hyperthermia of Chinese hamster ovary cells. *Cancer Res* **48**: 2417–2420

Dennis MF, Stratford, MRL, Wardman P and Watts ME (1985) Cellular uptake of misonidazole and analogues with acidic or basic functions. *Int J Radiat Biol* **47**: 629–643

Duchateau AMJA (1987) Mitoxantron (Novantrone). *Pharm Weekbl* **122**: 286–289

Fellenz MP and Gerweck LE (1988) Influence of extracellular pH on intracellular pH and cell energy status: relationship to hyperthermic sensitivity. *Radiat Res* **116**: 305–312

Gerweck LE and Seetharaman K (1996) The cellular pH gradient in tumor versus normal tissue: potential exploitation for the treatment of cancer. *Cancer Res* **56**: 1194–1198

Helmlinger G, Yuan F, Dellian M and Jain RK (1997) Interstitial pH and $pO_2$ gradients in solid tumors in vivo: high-resolution measurements reveal a lack of correlation. *Nature Med* **3**: 177–182

Jahde E, Glusenkamp K-H, Klunder I, Hulser DF, Tietze L-F and Rajewsky MF (1989) Hydrogen ion-mediated enhancement of cytotoxicity of bis-chlorethylating drugs in rat mammary carcinoma cells in vitro. *Cancer Res* **49**: 2965–2972

Jahde E, Glusenkamp K-H and Rajewsky MF (1990) Protection of cultured malignant cells from mitoxantrone cytotoxicity by low extracellular pH: a possible mechanism for chemoresistance in vivo. *Eur J Cancer* **26**: 101–106

Mikkelsen RB, Asher C and Hicks T (1985) Extracellular pH, transmembrane distribution and cytotoxicity of chlorambucil. *Biochem Pharmacol* **34**: 2531–2534

Parkins CS, Chadwick JA and Chaplin DJ (1996) Inhibition of intracellular pH control and relationship to cytotoxicity of chlorambucil and vinblastine. *Br J Cancer* **74** (suppl. XXVII): S75–S77

Roos A and Boron WF (1981) Inracellular pH. *Physiol Rev* **61**: 296–434

Skarsgard LD, Chaplin DJ, Wilson DJ, Skwarchuk MW, Vinczan A and Kristl J (1992) The effect of hypoxia and low pH on the cytotoxicity of chlorambucil. *Int J Radiat Oncol Biol Phys* **22**: 737–741

Skarsgard LD, Skwarchuk MW, Vinczan A, Kristl J and Chaplin D (1995) The cytotoxicity of melphalan and its relationship to pH, hypoxia and drug uptake. *Anticancer Res* **15**: 219–224

Thistlethwaite AJ, Alexander GA, Moylan DJ and Leeper DB (1987) Modification of human tumor pH by elevation of blood glucose. *Int J Radiat Oncol Biol Phys* **13**: 603–610.

Vaupel P, Kallinowski F and Okunieff P (1989) Blood flow, oxygen and nutrient supply, and metabolic microenvironment of human tumors: a review. *Cancer Res* **49**: 6449–6465

Vukovic V and Tannock IF (1997) Influence of low pH on cytotoxicity of paclitaxel, mitoxantrone and topotecan. *Br J Cancer* **75**: 1167–1172

Waddell WJ and Butler TC (1959) Calculation of intracellular pH from the distribution of 5,5-dimethyl-2,4-oxazolidinedione (DMO). Application to skeletal muscle of the dog. *J Clin Invest* **38**: 720–729

Wahl ML, Coss RA, Bobyock SB, Leeper DB and Owen CS (1996) Thermotolerance and intracellular pH in two Chinese hamster cell lines adapted to growth at low pH. *J Cell Physiol* **166**: 438–445

Wike-Hooley JL, Haveman J and Reinhold HS (1984) The relevance of tumour pH to the treatment of malignant disease. *Radiother Oncol* **2**: 343–366

© Cancer Research Campaign 1998